# Early Gestational Hepatic Lipidomic Profiles Are Modulated by One-Carbon Metabolite Supplementation and Nutrient Restriction in Beef Heifers and Fetuses

**DOI:** 10.3390/metabo15050302

**Published:** 2025-05-01

**Authors:** Kazi Sarjana Safain, Matthew S. Crouse, Jessica G. Syring, Yssi L. Entzie, Layla E. King, Alison K. Ward, Lawrence P. Reynolds, Pawel P. Borowicz, Carl R. Dahlen, Kendall C. Swanson, Joel S. Caton

**Affiliations:** 1 Department of Animal Sciences, Center for Nutrition and Pregnancy, North Dakota State University, Fargo, ND 58108, USA; jessica.syring@ndsu.edu (J.G.S.); yssi.entzie@ndsu.edu (Y.L.E.); larry.reynolds@ndsu.edu (L.P.R.); pawel.borowicz@ndsu.edu (P.P.B.); carl.dahlen@ndsu.edu (C.R.D.); kendall.swanson@ndsu.edu (K.C.S.); joel.caton@ndsu.edu (J.S.C.); 2 USDA, ARS, U.S. Meat Animal Research Center, Clay Center, NE 68933, USA; matt.crouse@usda.gov; 3 Department of Agriculture and Natural Resources, University of Minnesota Crookston, Crookston, MN 56716, USA; king0635@crk.umn.edu; 4 Department of Veterinary Biomedical Sciences, University of Saskatchewan, Saskatoon, SK S7N 5B4, Canada; alison.ward@usask.ca

**Keywords:** beef heifers, early gestation, fetal development, lipidomics, nutrient restriction, one-carbon metabolism

## Abstract

**Background:** Maternal nutrition during early gestation induces metabolic adaptations that support maternal health and fetal development. This study evaluated the effects of maternal one-carbon metabolite (OCM: methionine, choline, folate, and vitamin B_12_) supplementation and restricted rates of maternal gain on the hepatic lipid profiles of dams and fetuses at day 63 of gestation. **Methods:** Thirty-one crossbred Angus heifers were inseminated and assigned to a 2 × 2 factorial design with two factors: maternal dietary intake (control [CON]; 0.60 kg/day average daily gain [ADG] vs. restricted [RES]; −0.23 kg/day ADG) and OCM supplementation (supplemented [+OCM] vs. not supplemented [−OCM]). The four resulting groups (CON − OCM, CON + OCM, RES − OCM, RES + OCM) were maintained for 63 days post-breeding. Maternal and fetal liver samples were collected, and lipidomic profiling was performed using ultra-performance liquid chromatography–tandem mass-spectrometry. **Results:** In maternal liver, 485 lipid metabolites were detected, with 243 differing significantly in maternal gain. RES heifers showed increased levels (*p* ≤ 0.05) of acylcarnitines, plasmalogens, lysoplasmalogens, glycosphingolipids, and sphingomyelins. Additionally, RES combined with OCM supplementation led to the accumulation of secondary bile acids and a depletion of monoacylglycerols (*p* ≤ 0.05) in maternal liver. In fetal liver, 487 lipid metabolites were detected, but treatment effects were minimal. **Conclusions:** Maternal rate of gain significantly influenced hepatic lipid metabolism in the maternal liver, while fetal liver lipid profiles remained relatively unaffected. These findings underscore the significant role of dietary intake/rate of gain compared with OCM supplementation in modulating hepatic lipid metabolism and highlight the maternal liver’s metabolic adaptations during early pregnancy.

## 1. Introduction

Liver, the primary metabolic organ in mammals, plays a central role in regulating essential metabolic functions, including the storage and metabolism of amino acids, carbohydrates, bile acids, lipids (including cholesterol, fatty acids, etc.), proteins, and vitamins [1]. During pregnancy, the maternal liver undergoes significant metabolic adaptations to support the increased energy and nutrient demands of gestation [2]. These adaptations prioritize maternal energy reserves, predominantly in the form of lipids and proteins, to sustain maternal–fetal needs. While glucose and amino acids are the primary nutrients transferred to the fetus through the placenta, lipid transfer is relatively limited [3]. Despite the relatively small amount of transfer across the placenta, lipids play an important role in fetal development, as changes in maternal dietary lipid composition can influence fetal and postnatal growth [4]. The dysregulation of maternal lipid metabolism during pregnancy has been linked to adverse outcomes for both the mother and offspring [5], underscoring the importance of a tightly regulated lipid metabolism throughout gestation.

Beyond immediate fetal growth, maternal nutrition also affects fetal epigenetic programming [6], with implications for offspring health across the lifespan [6,7]. In livestock, these developmental programming effects influence fetal growth, postnatal performance, and overall productivity [8]. Early pregnancy is a critical period for these programming events, particularly in beef cattle, where nutrient availability can impact DNA methylation and other epigenetic mechanisms [9].

Early pregnancy is a pivotal period for reproductive efficiency and herd health in beef cattle [10]. This phase is shaped by developmental programming, where epigenetic mechanisms, such as DNA methylation and histone modifications, influence long-term postnatal outcomes. The establishment of these epigenetic marks depends on the availability of one-carbon metabolites (OCM), including, among others, methionine, choline, folate, and vitamin B_12_. A one-carbon metabolism encompasses interconnected pathways critical for DNA, RNA, and protein methylation, as well as other metabolic processes [11]. Due to the interconnected nature of one-carbon metabolism, imbalances in any component may disrupt metabolic homeostasis. Supplementing OCM during early gestation could mitigate the effects of nutrient restriction by supporting epigenetic and metabolic processes [12].

Previously, our research team investigated how maternal nutrient restriction and OCM supplementation influence maternal and fetal outcomes during the first trimester of gestation in beef cattle [13]. In that study, we focused on fetal liver metabolomics in pregnant beef heifers, highlighting alterations in key metabolic pathways, including amino acid, vitamin, carbohydrate, and energy metabolism. These findings revealed that maternal nutrient restriction reduced fetal liver metabolite concentrations, while OCM supplementation increased specific metabolites, indicating a potential protective role of OCM in supporting key developmental pathways. While the roles of some one-carbon metabolites in lipid metabolism are well-documented in dairy [14] and human studies [15], their specific implications for maternal and fetal lipid profiles in beef cattle remain less explored. Further investigation is therefore needed to understand how maternal OCM supplementation influences hepatic lipid metabolism in this context.

To address this knowledge gap, we conducted a comprehensive lipidomic analysis to characterize the maternal and fetal hepatic lipid profiles at day 63 of gestation in response to maternal OCM supplementation and the restricted rate of gain. We hypothesized that maternal OCM supplementation combined with restricted gain would result in significant alterations in maternal and fetal hepatic lipid metabolism, evident through changes in the abundance of key lipid classes. These findings will enhance our understanding of maternal–fetal nutrient transfer, lipid metabolism, and the role of maternal nutrition in fetal developmental programming during early gestation.

## 2. Materials and Methods

### 2.1. Animals and Ethics Statement

All experimental protocols were approved by the Animal Care and Use Committee at North Dakota State University. The study’s design, treatments, and dietary regimens are detailed in the work of Safain et al. [13]. In brief, 72 heifers, predominantly of Angus lineage, underwent estrous synchronization using a 7-day select Synch + CIDR protocol and were artificially inseminated with female-sexed semen from a single sire (Maternal Made [ST Genetics, Navasota, TX, USA]). Heifers were assigned to two targeted gain levels: control (CON: targeted at 0.45 kg/d ADG, achieved 0.60 kg/d ADG) and restricted (RES: targeted at −0.23 kg/d ADG, achieved −0.23 kg/d ADG). Diets were delivered daily to individual heifers in a Calan gate system as a total mixed ration including corn silage, alfalfa hay, corn grain, and alfalfa/grass hay, and were formulated and fed to achieve the desired gains. Additionally, a daily top-dressed vitamin and mineral premix (Trouw dairy VTM w/Optimins, Trouw Nutrition USA, Highland, IL, USA) was administered via a fine-ground corn carrier. For further details of the diet composition, refer to Syring et al. [16]. Body weight measurements were taken weekly, with diet delivery adjustments as needed to maintain the targeted ADG.

Heifers were divided within their targeted gain level to receive either OCM supplementation (+OCM; 7.4 g/d of rumen-protected methionine [Smartamine, Adisseo, Nanjing, China], 44.4 g/d of rumen-protected choline [ReaShure, Balchem Inc., New Hampton, NY, USA], 20 mg of vitamin B_12_ [MWI Animal Health, Boise, ID, USA], and 320 mg of folic acid [Spectrum Chemical Mfg. Corp., New Brunswick, NJ, USA]) or no OCM supplementation (−OCM; provided with corn carrier and saline injections). Methionine and choline were included in the daily fine-ground corn carrier. Folate (320 mg in 6 mL of saline), vitamin B_12_ (20 mg in 4 mL of saline), or saline (6 mL and 4 mL) were injected intramuscularly each week. The study followed a completely randomized design with a 2 × 2 factorial arrangement of treatments, with gain level and OCM supplementation as the main factors. The four dietary treatment groups were maintained for 63 days post-breeding: CON − OCM, CON + OCM, RES − OCM, and RES + OCM. Ultrasound assessments on day 63 determined fetal sex, and only pregnancies with female fetuses continued in the study [17]. This resulted in a final sample size of 31 pregnant heifers with female fetuses, distributed as follows: CON − OCM (*n* = 7), CON + OCM (*n* = 7), RES − OCM (*n* = 9), and RES + OCM (*n* = 8).

### 2.2. Sample Collection, Storage and Preparation

At day 63 of gestation, heifers were slaughtered at the USDA-inspected, NDSU Meat Laboratory. Sample collection, handling, and storage procedures were adapted following validated methodologies described by Crouse et al. [18]. Maternal and fetal liver samples were promptly snap-frozen in liquid nitrogen and stored at −80 °C. Metabolomic profiling of these liver samples was conducted by Metabolon (Metabolon, Inc., Durham, NC, USA). Sample preparation was automated using the MicroLab STAR^®^ system (Hamilton Company, Reno, NV, USA), with aliquots prepared for reverse phase UPLC-MS/MS and HILIC/UPLC-MS/MS analysis. Compound identification was carried out by comparing the results to a library of purified standards or recurrent unknown compounds, as described by Menezes et al. [19].

### 2.3. Quality Control

Quality control procedures, including the use of pooled matrix samples, process blanks, and internal standards, were implemented in accordance with best practices, as described by Mosley et al. [20]. To ensure data quality, several controls were included with the experimental samples, as follows: (1) a pooled matrix sample was analyzed repeatedly as a technical replicate across the dataset; (2) process blanks consisted of extracted water samples; and (3) each analyzed sample was spiked with a recovery standard and an internal standard to monitor instrument performance and ensure chromatographic alignment. Instrument variability was assessed by calculating the median relative standard deviation (RSD) for standards introduced into each sample prior to mass spectrometer injection. Process variability was determined by the median RSD for all endogenous metabolites (excluding instrument standards) present in 100% of the pooled matrix samples. The experimental samples were randomized throughout the analysis run, with QC samples interspersed at regular intervals. The mean instrument variability for internal standards was 3%, while the process variability for endogenous metabolites averaged 7%.

### 2.4. Ultrahigh-Performance Liquid Chromatography–Tandem Mass Spectroscopy (UPLC-MS/MS)

For metabolite analysis, a Waters ACQUITY UPLC system was coupled to a Thermo Scientific Q-Exactive, high-resolution/accurate mass spectrometer, equipped with a heated electrospray ionization (HESI-II) source and an Orbitrap mass analyzer operating at a resolution of 35,000. After drying, sample extracts were reconstituted in method-specific solvents, each containing a set of standards at consistent concentrations to maintain injection and chromatographic consistency. Untargeted UPLC-MS/MS analyses were performed based on validated high-resolution mass spectrometry methods, as outlined by Evans et al. [21].

### 2.5. Data Extraction and Compound Identification

Raw data were processed for peak extraction, compound identification, and quality control through Metabolon’s Laboratory Information Management System (LIMS). Compounds were identified by comparison to a reference library containing purified standards and recurrent unknown entities. Identification criteria included a narrow retention index window, precise mass matching within ±10 ppm, and forward and reverse MS/MS scores aligning with experimental data from authentic standards. Metabolites were semi-quantified using peak area-under-the-curve (AUC) values derived from MS signal intensities. These data reflect relative abundance but do not represent absolute concentrations or total pool sizes.

### 2.6. Statistical Analysis

Metabolite data were log-transformed, adjusted for liver weight, and analyzed via two-way ANOVA to assess the main effects of ADG, OCM supplementation, and their interaction. Contrasts between treatments were conducted using Welch’s two-sample *t*-tests. A significance level of *p* ≤ 0.05 was deemed significant, with tendencies noted for values between 0.05 and 0.10. Pathway enrichment analysis was performed with MetaboLync Pathway Analysis software (Metabolon, Inc., Morrisville, NC, USA), using the formula (k/m)/(*n*/N), where k is the number of significant metabolites in each pathway, m is the total number of metabolites identified in each pathway, *n* is the number of significant metabolites in the study, and N represents the total metabolites detected. The metabolites were grouped based on compound class, as defined by Metabolon’s internal classification system (e.g., lysophospholipids, dicarboxylates). “Pathway enrichment score” refers to statistical overrepresentation of compound classes (sub-pathways) based on Metabolon’s internal classification system and does not represent canonical metabolic pathways. This approach follows the method outlined by Simintiras et al. [22], with pathways showing enrichment scores greater than 1 indicating statistically significant fold-changes between treatments. Interpretations of pathway enrichment and metabolite abundance were based on relative changes in metabolite signal intensity, not absolute flux. Principal Component Analysis (PCA) was performed as an unsupervised dimensionality reduction method to identify patterns in lipidomic data. Each principal component was calculated as a linear combination of all measured metabolites, ensuring orthogonality between components. The first principal component captured the highest variance in the dataset, followed by subsequent components that maximized variance while remaining orthogonal to the preceding ones. The proportion of total variance explained by each component was computed to assess the contribution of different metabolic variations.

## 3. Results

### 3.1. Principal Component Analysis (PCA) of Lipid Profiles

**Figure 1:**
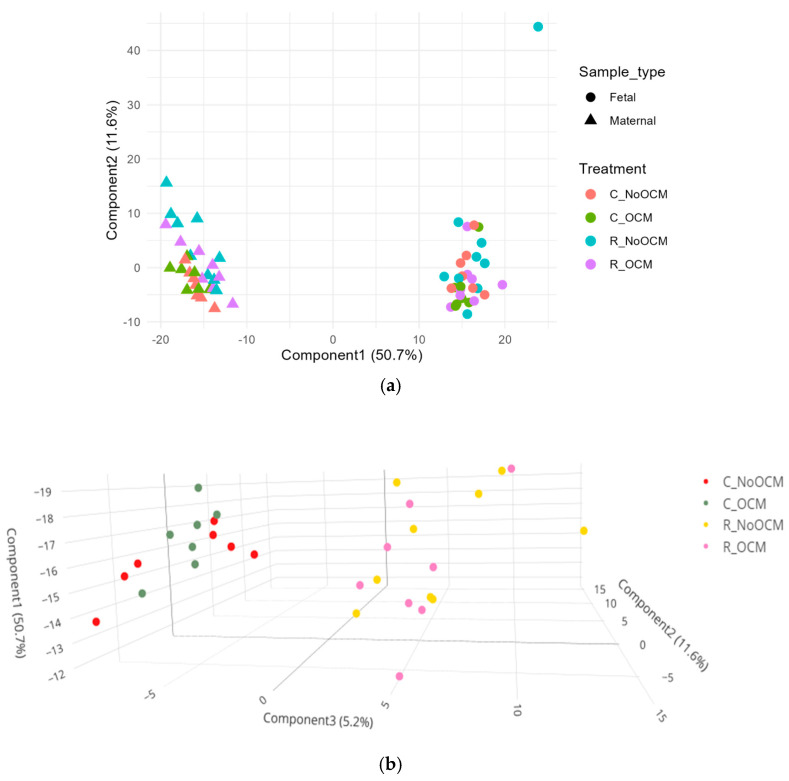

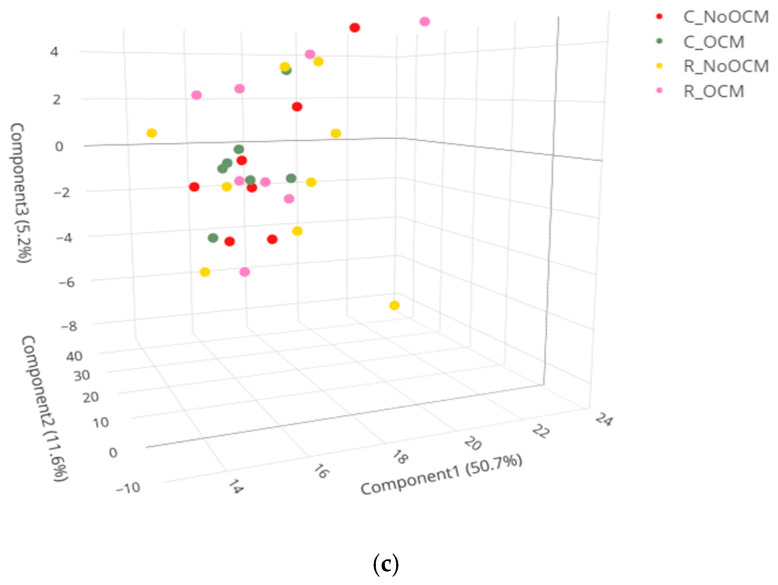
Principal component analysis (PCA) of lipid profiles in maternal and fetal liver across four treatment groups. (**a**) Combined PCA comparison of maternal and fetal liver lipid profiles. (**b**) PCA of lipid profiles in maternal liver categorized by treatment group. (**c**) PCA of lipid profiles in fetal liver categorized by treatment group. Treatment groups are defined as C (control) and R (restricted), with (OCM) or without OCM supplementation (NoOCM).

A PCA of fetal and maternal liver samples for lipid metabolites is presented in Figure 1. A clear separation between maternal and fetal liver samples was observed along the principal components, indicating distinct metabolic profiles for the two tissue types (Figure 1a). Maternal liver samples displayed noticeable clustering between the CON and RES groups, highlighting treatment-specific differences in lipid profiles for maternal liver (Figure 1b), whereas fetal liver samples did not show distinct clustering among the treatment groups (Figure 1c).

### 3.2. Metabolic Pathway Enrichment Analysis

All results, including *p*-values, fold changes, mean values, and percentage-filled values for all the analytes evaluated in this manuscript, are available in Supplementary File S1. Additionally, raw data and box plots for all the lipidomic analytes are provided in Supplementary File S2 and Supplementary Figure S1, respectively. In the context of pathway enrichment analysis, we identified a total of 53 sub-pathways for maternal liver and 55 sub-pathways for fetal liver based on the lipid profiles.

For fetal liver, several lipid metabolism sub-pathways were enriched under the Gain × OCM interaction (Table 1). These included fatty acid, dicarboxylate (1.93), fatty acid metabolism (acyl glycine; 6.53), primary bile acid metabolism (5.44), lysophospholipid (1.93), glycosyl PE (5.22), ceramides (1.31), phosphatidylglycerol (PG; 2.61), pregnenolone steroids (13.05), progestin steroids (13.05), fatty acid metabolism (acyl carnitine, polyunsaturated; 3.92), lysoplasmalogen (3.73), and phosphatidylethanolamine (PE; 2.18).

Regarding the main effect of OCM supplementation, several lipid metabolism sub-pathways were enriched in fetal liver (Table 1) including fatty acid, dicarboxylate (1.27), fatty acid metabolism (acyl glycine; 4.29), primary bile acid metabolism (4.29), fatty acid, monohydroxy (1.07), fatty acid metabolism (4.29), glycosyl PE (3.44), fatty acid, amino (4.29), sphingolipid synthesis (2.15), phosphatidylglycerol (PG; 5.15), fatty acid, branched (5.15), pregnenolone steroids (8.59), progestin steroids (8.59), long-chain polyunsaturated fatty acid (4.07), endocannabinoid (1.72), monoacylglycerol (3.62), long-chain saturated fatty acid (4.91), phosphatidylethanolamine (PE; 1.91), docosanoid (4.29), long-chain monounsaturated fatty acid (1.23), medium-chain fatty acid (8.59), dihydroceramides (4.29), and sphingosines (2.86).

Regarding the main effect of gain, enriched sub-pathways in fetal liver included fatty acid, dicarboxylate (2.45), fatty acid metabolism (acyl glycine; 3.97), primary bile acid metabolism (2.75), fatty acid, monohydroxy (1.10), fatty acid metabolism (3.31), glycosyl PE (2.64), fatty acid, amino (6.61), sphingolipid synthesis (1.65), fatty acid metabolism (acyl choline; 3.31), fatty acid metabolism (acyl carnitine, dicarboxylate; 2.20), mevalonate metabolism (2.20), phosphatidylglycerol (PG; 1.32), fatty acid, branched (1.32), ketone bodies (6.61), pregnenolone steroids (6.61), hexosylceramides (HCER; 3.31), and progestin steroids (6.61) (Table 1). All the remaining pathways had an enrichment score of less than 1 for the Gain × OCM, OCM, and Gain.

In the maternal liver, several lipid metabolism sub-pathways were enriched under the Gain × OCM interaction (Table 2). These included lysophospholipid (1.06), diacylglycerol (1.80), monoacylglycerol (3.41), long-chain polyunsaturated fatty acid (n3 and n6; 1.71), lysoplasmalogen (2.32), fatty acid metabolism (acyl carnitine, hydroxy; 2.03), fatty acid metabolism (acyl carnitine, polyunsaturated; 1.62), fatty acid metabolism (acyl carnitine, monounsaturated; 2.32), secondary bile acid metabolism (2.95), endocannabinoid (8.10), glycosyl PE (3.24), fatty acid, branched (3.24), and fatty acid metabolism (4.05). The remaining pathways had an enrichment score of less than 1 for the Gain × OCM interaction.

Regarding the main effect of OCM supplementation, several lipid metabolism sub-pathways were enriched in maternal liver (Table 2). These included sphingomyelins (1.04), diacylglycerol (1.01), monoacylglycerol (1.43), phospholipid metabolism (1.81), lysoplasmalogen (1.29), fatty acid metabolism (acyl carnitine, hydroxy; 1.13), fatty acid metabolism (acyl carnitine, monounsaturated; 1.29), phosphatidylserine (PS; 2.59), glycosyl PE (1.81), fatty acid metabolism (1.29), secondary bile acid metabolism (1.65), primary bile acid metabolism (1.51), sphingolipid synthesis (2.26), fatty acid metabolism (2.26), fatty acid, amino (4.53), inositol metabolism (4.53), and sphingosines (3.02).

Regarding the main effect of gain, the enriched sub-pathways in maternal liver included lysophospholipid (1.19), sphingomyelins (1.92), phosphatidylcholine (PC; 1.25), diacylglycerol (1.43), plasmalogen (1.26), monoacylglycerol (1.19), phosphatidylethanolamine (PE; 1.13), phosphatidylinositol (PI; 1.81), fatty acid metabolism (acyl carnitine, long-chain saturated; 2.26), phospholipid metabolism (1.36), lysoplasmalogen (1.94), fatty acid metabolism (acyl carnitine, hydroxy; 1.70), fatty acid metabolism (acyl carnitine, polyunsaturated; 1.13), fatty acid metabolism (acyl carnitine, monounsaturated; 1.62), phosphatidylserine (PS; 1.62), dihydrosphingomyelins (1.81), long-chain monounsaturated fatty acid (1.29), fatty acid metabolism (acyl carnitine, dicarboxylate; 2.26), phosphatidylglycerol (PG; 1.36), glycosyl PE (1.36), sterol (1.70), hexosylceramides (HCER; 2.26), fatty acid metabolism (acyl carnitine, short chain; 2.26), carnitine metabolism (1.13), fatty acid, oxidized (2.26), docosanoid (1.13), medium-chain fatty acid (2.26), dihydroceramides (1.13), lactosylceramides (LCER; 2.26), fatty acid, amino (1.13), inositol metabolism (1.13), and progestin steroids (2.26). The remaining pathways had an enrichment score of less than 1 for Gain, OCM and their interaction.

### 3.3. Statistical Analysis of Main Effects and Interactions in Fetal Liver

A total of 487 lipid metabolites were identified in fetal liver (Supplementary File S1). Among these, 15 metabolites were significantly influenced by the Gain × OCM interaction (*p* ≤ 0.05), with an additional 25 metabolites showing a tendency toward this effect (0.05 < *p* ≤ 0.10). The main effect of the rate of gain significantly affected 29 metabolites (*p* ≤ 0.05), and 17 metabolites exhibited a tendency for this effect (0.05 < *p* ≤ 0.10). OCM supplementation significantly influenced 57 metabolites (*p* ≤ 0.05), with a tendency observed for 38 additional metabolites (0.05 < *p* ≤ 0.10). For brevity, only the most significantly altered sub-pathways and metabolites are highlighted in the following discussion.

Among the metabolites in the fatty acid metabolism (acyl carnitine) pathways (short-chain, medium-chain and long-chain saturated, monounsaturated, polyunsaturated, dicarboxylate, and hydroxy); dihomo-linoleoylcarnitine and docosatrienoylcarnitine were affected by a Gain × OCM interaction (*p* = 0.04; Table 3). The highest levels of dihomo-linoleoylcarnitine were observed in the RES + OCM group compared to all other treatments. Additionally, cis-4-decenoylcarnitine and pimeloylcarnitine/3-methyladipoylcarnitine were greater in RES compared to CON (*p* ≤ 0.04). None of the metabolites in these pathways were affected by the main effects of OCM (*p* ≥ 0.11; Supplementary File S1).

In the plasmalogen and lysoplasmalogen metabolism pathways, 1-palmityl-GPC and 1-stearyl-GPC were influenced by the Gain × OCM interaction (*p* ≤ 0.03; Table 3), with levels ~1.45-fold greater in CON + OCM compared to other treatments. The only metabolite influenced by gain was 1-palmityl-2-palmitoyl-GPC from the plasmalogen pathway, with levels 1.11-fold greater in RES compared to CON (*p* = 0.02). No metabolites in this pathway were significantly affected by OCM (*p* ≥ 0.10; Supplementary File S1).

None of the monoacyl- and diacylglycerols were influenced by the Gain × OCM interaction (*p* ≥ 0.10; Supplementary File S1); however, several monoacylglycerols, including 1-myristoylglycerol, 1-pentadecanoylglycerol, 1-linoleoylglycerol, 1-docosahexaenoylglycerol, 2-myristoylglycerol, 2-docosahexaenoylglycerol, 1-heptadecenoylglycerol, and 2-heptadecenoylglycerol, were greater in −OCM than +OCM (*p* ≤ 0.05; Table 3). Among the diacylglycerols, only linoleoyl-docosahexaenoyl-glycerol was affected by OCM, with levels greater in −OCM than in +OCM (*p* ≤ 0.03).

No hexosylceramides or lactosylceramides were affected by either the Gain × OCM interaction (*p* ≥ 0.75; Supplementary File S1) or by the main effect of OCM (*p* ≥ 0.81); however, the levels of glycosyl-N-stearoyl-sphingosine were greater in RES than in CON (*p* = 0.02; Table 3).

Similarly, none of the sphingomyelins were influenced by the Gain × OCM interaction (*p* ≥ 0.16; Supplementary File S1) or the main effect of gain (*p* ≥ 0.12). The only sphingomyelin significantly affected by OCM was sphingomyelin (d18:2/23:1), which had greater concentrations in −OCM than in +OCM (*p* = 0.03; Table 3).

The following sub-pathways showed no metabolites affected by the Gain × OCM interaction or the main effects of gain and OCM (*p* ≥ 0.05; Supplementary File S1): fatty acid synthesis, fatty acid metabolism, fatty acid metabolism (also BCAA Metabolism), fatty acid metabolism (acyl carnitine, short chain), fatty acid metabolism (acyl carnitine, medium-chain), fatty acid metabolism (acyl carnitine, long-chain saturated), fatty acid metabolism (acyl carnitine, hydroxy), carnitine metabolism, fatty acid, dihydroxy, eicosanoid, inositol metabolism, glycosyl pe, phosphatidylserine, glycerolipid metabolism, dihydrosphingomyelins, sterol, pregnenolone steroids, progestin steroids and secondary bile acid metabolism.

### 3.4. Statistical Analysis of Main Effects and Interactions in Maternal Liver

A total of 485 metabolites were identified in maternal liver, among which 18 metabolites were significantly influenced by the Gain × OCM interaction (*p* ≤ 0.05; Table 4) and 16 metabolites showed a tendency toward this interaction (0.05 < *p* ≤ 0.10). The main effect of the rate of gain significantly affected 243 metabolites (*p* ≤ 0.05), with a tendency observed in 38 additional metabolites (0.05 < *p* ≤ 0.10). OCM supplementation significantly influenced 24 metabolites (*p* ≤ 0.05), with 16 metabolites tending toward significance (0.05 < *p* ≤ 0.10). For brevity, only the most significantly altered sub-pathways and metabolites are highlighted below.

The metabolites within the short-, medium-, and long-chain saturated acylcarnitine pathways were not significantly affected by the Gain × OCM interaction (*p* ≥ 0.06; Supplementary File S1); however, significant main effects of gain were observed across all these pathways. For the acylglycine pathway, valerylglycine, N-palmitoylglycine, and N-oleoylglycine were significantly more abundant in RES than in CON (*p* = 0.01; Table 4). Similarly, all short-, medium-, and long-chain saturated acylcarnitines, except decanoylcarnitine, showed significantly greater concentrations in RES compared to CON (*p* = 0.01).

In the mono- and polyunsaturated acylcarnitine pathways, only eicosenoylcarnitine (acyl carnitine, monounsaturated) was significantly influenced by the Gain × OCM interaction (*p* = 0.01; Table 4), with 2.24-fold and 0.54-fold greater levels in RES − OCM compared to CON − OCM and RES + OCM, respectively. This metabolite was also influenced by the main effect of OCM, with greater concentrations in −OCM than +OCM (*p* = 0.01). Additionally, all monounsaturated acylcarnitines, except cis-4-decenoylcarnitine and nervonoylcarnitine, were more abundant in RES than CON (*p* = 0.01). Among the polyunsaturated acylcarnitines, linoleoylcarnitine (C18:2), linolenoylcarnitine (C18:3), and arachidonoylcarnitine (C20:4) were significantly more abundant in RES than CON (*p* = 0.01), while adrenoylcarnitine was more abundant in CON compared to RES (*p* = 0.01).

Within the dicarboxylate and hydroxy acylcarnitine pathways, none of the metabolites were significantly influenced by the Gain × OCM interaction (*p* ≥ 0.47; Supplementary File S1) or by the main effect of OCM (*p* ≥ 0.32); however, the main effect of gain significantly affected nearly all metabolites, except for 3-hydroxyhexanoylcarnitines. For the remaining metabolites in these pathways, concentrations were greater in RES compared to CON (*p* = 0.01; Table 4).

In the plasmalogen and lysoplasmalogen pathway, no metabolites were significantly affected by the Gain × OCM interaction (*p* ≥ 0.13; Supplementary File S1) or the main effect of OCM (*p* ≥ 0.34). Several metabolites were influenced by the main effect of gain: 1-palmityl-2-palmitoyl-GPC, 1-palmityl-2-stearoyl-GPC, 1-palmityl-2-linoleoyl-GPC, 1-stearyl-2-arachidonoyl-GPC, 1-(1-enyl-palmitoyl)-2-linoleoyl-GPE, 1-(1-enyl-palmitoyl)-2-palmitoyl-GPC, 1-(1-enyl-palmitoyl)-2-arachidonoyl-GPE, 1-(1-enyl-stearoyl)-2-oleoyl-GPE and 1-(1-enyl-stearoyl)-2-arachidonoyl-GPE from Plasmalogen pathway (*p* = 0.01) and; 1-palmityl-GPC, 1-stearyl-GPC, 1-stearyl-GPE, 1-(1-enyl-palmitoyl)-GPE, and 1-(1-enyl-oleoyl)-GPE from the Lysoplasmalogen pathway (*p* = 0.01; Table 4) were influenced by the main effect of gain. With the exception of 1-(1-enyl-palmitoyl)-2-linoleoyl-GPE, all other metabolites’ concentrations were greater in RES than in CON (*p* = 0.01).

Among the monoacylglycerols, 1-linoleoylglycerol, 2-linoleoylglycerol, 2-arachidonoylglycerol, and 2-docosahexaenoylglycerol were influenced by the Gain × OCM interaction (*p* ≤ 0.03; Table 4), with these four metabolites showing the highest concentrations in RES − OCM. Further, OCM supplementation resulted in lower abundances of 1-docosahexaenoylglycerol, 2-myristoylglycerol, and 2-docosahexaenoylglycerol compared to the +OCM group (*p* ≤ 0.04). Additionally, the main effect of gain influenced several metabolites, including 1-myristoylglycerol, 1-palmitoleoylglycerol, 1-oleoylglycerol, 1-linoleoylglycerol, 2-oleoylglycerol, 2-linoleoylglycerol, 2-arachidonoylglycerol, 2-docosahexaenoylglycerol, 1-heptadecenoylglycerol, and 2-heptadecenoylglycerol, all of which were more abundant in RES compared to CON (*p* ≤ 0.05).

In the diacylglycerols, no significant effects of the Gain × OCM interaction (*p* ≥ 0.13; Supplementary File S1) or the main effect of OCM (*p* ≥ 0.11) were observed; however, restricted gain resulted in an increase in the accumulation of several diacylglycerols in RES compared to CON (*p* = 0.01; Table 4).

Among the hexosylceramides and lactosylceramides identified in maternal liver, no significant Gain × OCM interaction (*p* ≥ 0.13; Supplementary File S1) or main effect of OCM (*p* ≥ 0.33) were observed; however, all identified hexosylceramides and lactosylceramides were more abundant in RES compared to CON (*p* = 0.01; Table 4).

In the dihydrosphingomyelin and sphingomyelin pathways, no significant Gain × OCM interaction was observed (*p* ≥ 0.18; Supplementary File S1). Among the dihydrosphingomyelins, all but one metabolite were more abundant in RES than in CON (*p* = 0.01; Table 4). Two sphingomyelins were more abundant in −OCM than in +OCM (*p* ≤ 0.04). Further, 21 out of the 26 identified sphingomyelins were affected by the main effects of gain (*p* ≤ 0.04), showing greater abundance in RES than CON (*p* ≤ 0.02).

Among the sterols identified in maternal liver, no significant effects of the Gain × OCM interaction (*p* ≥ 0.13; Supplementary File S1) or the main effect of OCM (*p* ≥ 0.20) were observed. RES resulted in the accumulation of cholesterol, 4-cholesten-3-one, and 7-hydroxycholesterol, all of which were more abundant in RES compared to CON (*p* ≤ 0.04; Table 4).

In the secondary bile acid metabolism pathway, glycoursodeoxycholate, tauroursodeoxycholate, and taurocholenate sulfate were significantly influenced by the Gain × OCM interaction (*p* ≤ 0.05; Table 4). Glycoursodeoxycholate and tauroursodeoxycholate were 9.29-fold and 4.13-fold more abundant in RES + OCM than in all other treatment groups (*p* ≤ 0.03), respectively. Additionally, the main effect of gain significantly influenced glycoursodeoxycholate and tauroursodeoxycholate, which were 5.72-fold and 3.5-fold more abundant in RES compared to CON, respectively (*p* = 0.01). The main effect of OCM influenced taurodeoxycholate and glycocholenate sulfate, with the former being more abundant and the latter being less abundant in −OCM compared to +OCM (*p* ≤ 0.03).

**Figure 2:**
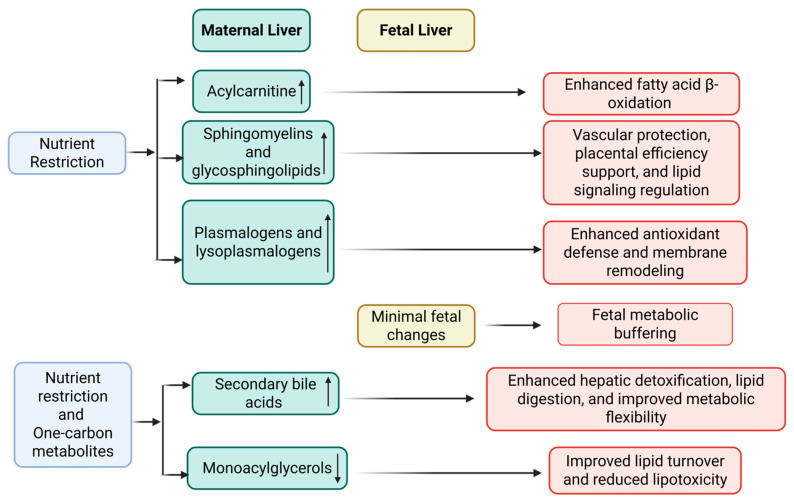
Summary of maternal and fetal hepatic lipid adaptations to nutrient restriction and one-carbon metabolite supplementation during early gestation. Arrows indicate direction of change in metabolite abundance relative to the control group: ↑ = increased; ↓ = decreased.

The following four sub-pathways showed no significant influence from the Gain × OCM interaction or the main effects of gain and OCM (*p* ≥ 0.10; Supplementary File S1): fatty acid synthesis, fatty acid metabolism, ketone bodies, and fatty acid metabolism (acyl choline). Additionally, Figure 2 summarizes the main hepatic lipidomic alterations and their physiological significance, as observed in maternal and fetal livers in response to nutrient restriction and one-carbon metabolite supplementation.

## 4. Discussion

In this lipidomic study, we built upon our previous findings [13] demonstrating the effects of maternal OCM supplementation and rates of gain (from breeding to day 63 of gestation) on the maternal and fetal liver lipidome. This study offered further insights through the quantitative profiling of 487 metabolites spanning 55 sub-pathways within the lipid metabolism super-pathway. For maternal liver, the majority of the metabolites (*n* = 243) were significantly influenced by the main effect of Gain, with greater abundances observed in RES liver compared to CON liver, whereas fetal liver lipid profiles remained relatively stable. This suggests that during early gestation, the maternal liver is the primary site for metabolic adaptation, ensuring adequate nutrient storage and biosynthesis to support pregnancy. In contrast, fetal liver responses appear buffered, possibly due to protective homeostatic mechanisms that maintain a stable intrauterine environment.

Our findings demonstrated that nutrient restriction/Gain led to distinct alterations in lipid classes in maternal liver. Specifically, there were increased concentrations of lysoplasmalogen, monoacylglycerol, diacylglycerol, hexosylceramides (HCER), lactosylceramides (LCER), dihydrosphingomyelins, sphingomyelins, and sterols, alongside decreased concentrations of branched fatty acids and phospholipids. Given that S-adenosylmethionine (SAM), the universal methyl donor, is synthesized via the one-carbon metabolism pathway—dependent on folate, vitamin B_12_, choline, and methionine—it is likely that the observed modulation of phospholipid metabolism, including phosphatidylcholine and lysophospholipids, was driven by the increase in SAM availability due to OCM supplementation. These lipid shifts reflect physiological adaptations aimed at enhancing fatty acid oxidation, protecting against oxidative stress, and maintaining membrane integrity during metabolic stress. Specifically, acylcarnitines serve as indicators of increased fatty acid β-oxidation [23], likely reflecting the liver’s compensatory mechanisms to meet energy demands. Elevated sphingomyelins and glycosphingolipids suggest enhanced signaling and vascular support [24], while increased plasmalogens and lysoplasmalogens contribute to antioxidative defenses and membrane remodeling [25].

In the fetal liver, OCM supplementation decreased the levels of several fatty acids, including long-chain saturated and polyunsaturated fatty acids, suggesting a potential role of OCM in modulating fetal lipid composition and improving metabolic efficiency. However, the extent of these changes was limited, emphasizing the fetus’s reliance on maternal metabolic buffering during early gestation. Additionally, the combined effects of OCM supplementation and RES revealed distinct lipidomic responses. In maternal liver, monoacylglycerols were decreased and secondary bile acids were increased under RES + OCM conditions. This suggests improved lipid turnover and enhanced bile-mediated lipid processing, possibly driven by methylation-dependent pathways supported by OCM availability. These results support the hypothesis that OCM supplementation under restricted gain enhances metabolic flexibility, contributing to more efficient hepatic lipid management.

Our current lipidomic findings showed that most lipid metabolites were elevated in RES compared to CON in the maternal liver, suggesting a coordinated metabolic adaptation driven by OCM to mitigate the effects of restricted nutrient availability. A similar phenomenon was observed in dairy cattle during the periparturient phase, where cows experience a negative energy balance due to high metabolic demands and reduced dry matter intake [26]. This period is marked by elevated non-esterified fatty acids and altered hepatic lipid metabolism, ultimately impacting milk production and metabolic efficiency. To counteract these effects, supplementation with rumen-protected methionine and choline has been shown to enhance nitrogen utilization, support lipid metabolism, and improve overall metabolic efficiency in lactating dairy cows [27]. Methionine plays a crucial role in methylation reactions and sulfur-containing amino acid synthesis, while choline supports DNA methylation, cell membrane stability, and lipid transport [28]. These mechanisms contribute to improved lipid mobilization, hepatic function, and lactation performance, while also mitigating the environmental impact of nitrogen excretion in dairy production. Although beef cattle do not experience the same extreme metabolic demands as lactating dairy cows, our RES model may induce comparable metabolic adaptations in the maternal liver due to restricted nutrient availability. These metabolic changes are likely mediated by one-carbon metabolism, as evidenced by our previous findings where RES increased maternal serum concentrations of vitamin B_12_ and hepatic levels of 5,10-methylenetetrahydrofolate and 5,10-methenyltetrahydrofolate [16]. These metabolites are key cofactors in one-carbon metabolism, which plays a central role in methylation-dependent processes, lipid biosynthesis, and turnover. Specifically, methylation pathways involving S-adenosylmethionine (SAM) are essential for phosphatidylcholine synthesis and lipid remodeling. Phosphatidylcholine synthesis, supported by methionine- and choline-derived methyl donors, is critical for maintaining membrane structure and lipoprotein secretion [29]. The observed increases in lipid classes such as sphingomyelins, diacylglycerols, and sterols in RES further support the hypothesis that one-carbon metabolite availability upregulates lipid metabolism in response to nutrient restriction. This aligns with findings in dairy cattle, where methionine and choline supplementation promote lipid mobilization and hepatic lipid metabolism, ultimately facilitating metabolic adaptations to support reproductive and physiological demands [27,30]. These results suggest that OCM-driven pathways play a mechanistic role in coordinating hepatic lipid metabolism under nutrient restriction, linking dietary intervention with lipid signaling and membrane lipid remodeling.

Our findings demonstrate that nutrient restriction triggers significant metabolic adjustments in the maternal liver, with 262 out of 485 metabolites (54%) being affected. In contrast, the fetal liver remained relatively less affected at this stage of gestation, with only 96 out of 487 metabolites (19%) influenced by maternal rate of gain, OCM supplementation, or their interaction. This disparity suggests that the fetal liver is shielded from maternal metabolic changes, likely due to the prioritization of nutrient partitioning mechanisms that preserve fetal organ development. Thus, the maternal liver serves as the primary site of metabolic adaptation, ensuring sufficient nutrient storage and metabolic preparation for later gestational demands. These findings align with human studies, where early gestation is characterized by an anabolic phase that prioritizes nutrient accumulation and metabolic preparation in the maternal liver for fetal energy demands later in pregnancy [31]. During this phase, lipid metabolism adapts to store energy, whereas in late gestation, the shift to a catabolic phase involves increased lipolysis, the mobilization of triglycerides, and elevated levels of very low-density lipoprotein (VLDL) and its remnants, driven by decreased lipoprotein lipase activity [32]. These metabolic shifts highlight the maternal body’s dynamic adjustments across gestation stages. Although our analysis was limited to the first trimester, these findings highlight the necessity for further lipidomic studies spanning subsequent stages of gestation to comprehensively characterize metabolic shifts and fetal adaptations across the entire gestational timeline.

Moreover, in this study, the limited changes observed in fetal hepatic metabolites suggest a prioritization of nutrient partitioning during early gestation, ensuring the protection of critical fetal organs such as the brain, heart, and liver. These metabolic adjustments align with studies reporting that maternal consumption of 70% of the global ad libitum diet during early pregnancy results in an 11% decrease in maternal body weight without significantly affecting fetal growth or the weights of essential organs like the liver or kidney [33]. Such findings collectively support the maternal system’s ability to mobilize and reallocate energy stores under nutrient restriction to safeguard fetal development; however, such adaptations can only effectively compensate within certain metabolic limits. The limited fetal response observed in our study further supports the concept that early gestation is an anabolic phase where maternal metabolic adjustments play a pivotal role in sustaining pregnancy and preparing for future fetal energy demands.

Our results indicate that the maternal liver of RES, compared with CON, had greater abundances of acylcarnitines (Fatty Acid Metabolism [Acyl Carnitine, Short-, Medium-, and Long-Chain], and Fatty Acid Metabolism [Acyl Carnitine, Monounsaturated]). Acylcarnitines, also referred to as fatty acid–carnitine esters, are a group of lipids (predominantly C2–C26) produced through fatty acid β-oxidation (FAO) [34], a crucial biochemical pathway for energy generation from fats [35]. Research on pregnant women has shown that elevated acylcarnitine levels are linked to gestational diabetes mellitus and increased risk of obesity in their offspring during childhood [36]. Additionally, increased acylcarnitine concentrations have been observed in obese individuals; their accumulation may disrupt insulin signaling and contribute to the development of insulin resistance [37]. Our findings that RES had more acylcarnitines in maternal liver thus indicate that restricted nutrition may enhance fatty acid oxidation, likely as an adaptive mechanism to meet energy demands under nutrient-limited conditions. Elevated acylcarnitines in RES may reflect a heightened reliance on lipid mobilization and oxidation to compensate for insufficient glucose availability, as seen in other states of metabolic stress. This adaptation, while beneficial for maintaining maternal energy balance, may have implications if prolonged, as excessive acylcarnitine accumulation has been associated with mitochondrial overload and metabolic dysfunction [38]. The elevated levels of acylcarnitines in RES suggest a potential metabolic trade-off: while facilitating immediate energy needs through enhanced FAO, prolonged nutrient restriction could predispose the dam and offspring to metabolic complications, including impaired insulin signaling or oxidative stress.

Sphingolipids are critical constituents of cellular membranes and play pivotal roles in lipid signaling, apoptosis, and maintaining cellular homeostasis [39]. It has been demonstrated that sphingolipids, including sphingomyelins, undergo significant alterations during pregnancy, with elevated levels reported in maternal plasma compared to postpartum [40]. These changes are vital for the physiological adaptations required for vascular endothelial function, lipid metabolism, and fetal development. Additionally, sphingolipids, particularly sphingomyelins, support vascular integrity and placental function by modulating vascular endothelial cell activity and lipid transport during pregnancy [41].

In the current study, Gain significantly affected the abundance of sphingomyelins in RES compared to CON maternal liver, with greater concentrations observed in the RES group. This finding reflects the maternal liver’s metabolic adjustments under nutrient restriction, potentially to support vascular health and thus placental efficiency. These lipids may serve as signaling intermediates linking maternal lipid metabolism with placental vascular development. Importantly, studies on sphingolipid metabolism in mice highlight the role of sphingosine kinase enzymes in producing sphingosine-1-phosphate (S1P), a key signaling lipid that regulates angiogenesis and vascular remodeling during pregnancy. Disruptions in this pathway resulted in impaired placental vascular development and early pregnancy loss, emphasizing the importance of sphingolipids in sustaining pregnancy [42]. These findings underscore the potential significance of increased sphingomyelins in RES maternal liver as a compensatory mechanism to support hepatic lipid signaling and maintain systemic and placental vascular and metabolic homeostasis during nutrient restriction.

Plasmalogens and lysoplasmalogens are critical phospholipids that support liver function, both by contributing to membrane stability and through offering protection against oxidative stress [43]. Plasmalogens, with their characteristic vinyl–ether bond, serve as potent antioxidants [44], while lysoplasmalogens, formed via the enzymatic cleavage at the sn-2 position, act as important intermediates in plasmalogen metabolism and can reflect active lipid remodeling [45]. Reports in the literature have consistently linked greater levels of these lipids to enhanced hepatic resilience and efficient lipid metabolism [46]. In our study, RES demonstrated increased levels of both plasmalogen and lysoplasmalogen compared to CON in maternal liver at day 63 of gestation. These findings suggest that nutrient restriction may upregulate plasmalogen and lysoplasmalogen synthesis or reduce their turnover, thereby providing a protective metabolic adaptation to maintain liver health during early gestation.

A combination of restricted gain and OCM supplementation resulted in lower concentrations of monoacylglycerols (MAGs) in the maternal liver. MAGs are intermediate products in triglyceride metabolism and play a key role in lipid signaling pathways [47], which are crucial for maintaining energy homeostasis and supporting the increased metabolic demands of pregnancy. Interestingly, bile acids such as glycoursodeoxycholate and tauroursodeoxycholate were found to be significantly elevated in RES + OCM, with concentrations that were 9-fold and 4.13-fold greater, respectively, compared to other treatments. Both of these conjugated bile acids are critical for lipid digestion, absorption, and metabolic regulation [48], and their marked increase suggests a heightened capacity for bile acid-mediated lipid processing under RES + OCM conditions. These elevations may reflect an adaptive mechanism to optimize nutrient absorption and energy extraction in response to restricted dietary intake, ensuring that lipid metabolism remains sufficient to meet both maternal and fetal demands. Elevated tauroursodeoxycholate, in particular, has been linked to protective roles against metabolic stress and may contribute to maintaining hepatic function during energy-restricted states [49]. Similarly, the increase in glycoursodeoxycholate might enhance lipid emulsification and transport [50], aligning with the observed decrease in MAG concentrations, which likely reflects improved lipid turnover and reduced lipotoxic intermediate accumulation. These observations align with findings from previous studies where maternal dietary energy intake influenced lipid profiles in fetal liver. Specifically, lower maternal rates of gain (0.28 kg/d) were associated with reduced MAG concentrations, reflecting adaptations in lipid remodeling and oxidation pathways during early gestation [19]. Similarly, lower MAG concentrations in our study may indicate a reduced accumulation of lipotoxic intermediates, favoring hepatic lipid utilization and storage pathways, particularly in the context of nutrient partitioning and fetal programming. These findings suggest that OCM supplementation—especially folate and choline—may facilitate enhanced bile acid synthesis via their role in methyl-group transfer and hepatic detoxification, possibly explaining the elevated tauroursodeoxycholate and glycoursodeoxycholate in the RES + OCM group. The concurrent availability of one-carbon metabolites likely supports these metabolic shifts by enhancing the methylation-dependent pathways involved in lipid metabolism [51], linking OCM to energy-efficient lipid utilization. These findings highlight the complex interplay between maternal nutrition, bile acid metabolism, and hepatic lipid homeostasis during early gestation, ensuring adequate energy supply and metabolic efficiency for both maternal and fetal demands.

This study has several limitations that should be acknowledged. First, the investigation was conducted at a single time point corresponding to early gestation (day 63), and lipid metabolism may undergo further adaptations during mid- to late gestation that were not captured. Second, the study focused exclusively on beef heifers of predominantly Angus lineage, which may limit the generalizability of the findings to other bovine breeds or species. Third, lipidomic profiling was based on the relative quantification of metabolite abundance and did not include direct measurements of absolute metabolite concentrations, metabolic fluxes, or enzyme activities. Finally, although the sample size was adequate to detect major treatment effects, smaller differences may have gone undetected. Future studies incorporating longitudinal sampling, stable isotope-based flux analysis, and larger populations across multiple breeds will be important to fully characterize the metabolic adaptations induced by maternal nutrition and one-carbon metabolite supplementation across gestation.

Overall, our findings partially support the original hypothesis. While maternal OCM supplementation combined with restricted gain significantly altered hepatic lipid metabolism in the maternal liver, changes in the fetal liver lipidome were relatively limited at this early stage of gestation. This suggests that the maternal liver undergoes marked metabolic adaptations under nutrient restriction and OCM supplementation, whereas the fetal liver may be protected from such changes during early development. These results emphasize the maternal liver’s central role in coordinating metabolic responses to dietary interventions during the first trimester of pregnancy.

## 5. Conclusions

Our comprehensive lipidomic analysis demonstrated that maternal lipid metabolism during early gestation was highly responsive to restricted nutrient intake, with widespread alterations observed in key lipid classes such as acylcarnitines, sphingomyelins, and plasmalogens. These changes suggest metabolic adaptations that may support maternal energy balance and hepatic function under nutrient-limited conditions in beef cattle. Further, a combination of OCM supplementation and restricted gain resulted in lower concentrations of monoacylglycerols and an increased concentration of some secondary bile acids, suggesting improved lipid metabolism and the reduced accumulation of intermediate lipid species. These alterations in lipid concentrations may also reflect a more efficient lipid utilization pathway, potentially driven by methylation-dependent mechanisms facilitated by OCM supplementation. In contrast, fetal hepatic lipid profiles remained largely unaffected at this stage, pointing to maternal buffering mechanisms that preserve fetal metabolic stability. Collectively, these findings highlight the dynamic nature of maternal hepatic lipid metabolism during early gestation, demonstrating how maternal nutritional strategies, including rate of gain and OCM supplementation, can significantly alter lipid profiles while maintaining fetal lipid homeostasis at this stage. These insights provide a foundation for understanding how maternal nutrition shapes metabolic adaptations during pregnancy and may inform targeted dietary strategies to optimize gestational outcomes, warranting further studies to determine the persistence of these effects and their long-term implications for maternal and offspring health.

## Figures and Tables

**Table 1:** Pathway enrichment scores for each pathway identified in fetal liver. Pathways with enrichment scores >1 have more metabolites with statistically significant fold changes compared to all other pathways within the study.

Sub-Pathway	Pathway Enrichment Score ^1^		
	Gain	OCM	Gain × OCM
Fatty Acid, Dicarboxylate	2.45	1.27	1.93
Fatty Acid Metabolism (Acyl Glycine)	3.97	4.29	6.53
Primary Bile Acid Metabolism	2.75	4.29	5.44
Fatty Acid, Monohydroxy	1.10	1.07	0.00
Lysophospholipid	0.22	0.84	1.93
Phosphatidylcholine (PC)	0.35	0.68	0.34
Fatty Acid Metabolism	3.31	4.29	6.53
Glycosyl PE	2.64	3.44	5.22
Fatty Acid, Amino	6.61	4.29	0.00
Phosphatidylinositol (PI)	0.66	0.00	0.00
Sphingolipid Synthesis	1.65	2.15	0.00
Fatty Acid Metabolism (Acyl Choline)	3.31	0.00	0.00
Ceramides	0.66	0.00	1.31
Diacylglycerol	0.24	0.64	0.48
Fatty Acid Metabolism (Acyl Carnitine, Dicarboxylate)	2.20	0.00	0.00
Plasmalogen	0.37	0.00	0.00
Mevalonate Metabolism	2.20	0.00	0.00
Sphingomyelins	0.25	0.33	0.00
Phosphatidylglycerol (PG)	1.32	5.15	2.61
Phospholipid Metabolism	0.66	0.86	0.00
Fatty Acid Metabolism (Acyl Carnitine, Monounsaturated)	0.94	0.00	0.00
Fatty Acid, Branched	1.32	5.15	0.00
Ketone Bodies	6.61	0.00	0.00
Pregnenolone Steroids	6.61	8.59	13.05
Hexosylceramides (HCER)	3.31	0.00	0.00
Progestin Steroids	6.61	8.59	13.05
Fatty Acid, Dihydroxy	0.00	0.00	0.00
Dihydrosphingomyelins	0.00	0.00	0.00
Long-Chain Polyunsaturated Fatty Acid (n3 and n6)	0.00	4.07	0.00
Endocannabinoid	0.00	1.72	0.00
Carnitine Metabolism	0.00	0.00	0.00
Fatty Acid Metabolism (Acyl Carnitine, Medium-Chain)	0.00	0.00	0.00
Eicosanoid	0.00	0.00	0.00
Secondary Bile Acid Metabolism	0.00	0.00	0.00
Monoacylglycerol	0.00	3.62	0.00
Glycerolipid Metabolism	0.00	0.00	0.00
Fatty Acid Metabolism (Acyl Carnitine, Polyunsaturated)	0.00	0.00	3.92
Lysoplasmalogen	0.00	0.00	3.73
Long-Chain Saturated Fatty Acid	0.00	4.91	0.00
Phosphatidylethanolamine (PE)	0.00	1.91	2.18
Fatty Acid, Oxidized	0.00	0.00	0.00
Sterol	0.00	0.00	0.00
Docosanoid	0.00	4.29	0.00
Long-Chain Monounsaturated Fatty Acid	0.00	1.23	0.00
Phosphatidylserine (PS)	0.00	0.00	0.00
Medium-Chain Fatty Acid	0.00	8.59	0.00
Dihydroceramides	0.00	4.29	0.00
Lactosylceramides (LCER)	0.00	0.00	0.00
Fatty Acid Metabolism (also BCAA Metabolism)	0.00	0.00	0.00
Fatty Acid Metabolism (Acyl Carnitine, Long-Chain Saturated)	0.00	0.00	0.00
Fatty Acid Metabolism (Acyl Carnitine, Hydroxy)	0.00	0.00	0.00
Sphingosines	0.00	2.86	0.00
Fatty Acid Metabolism (Acyl Carnitine, Short-Chain)	0.00	0.00	0.00
Inositol Metabolism	0.00	0.00	0.00
Fatty Acid Synthesis	0.00	0.00	0.00

^1^ Pathway enrichment was calculated within the MetaboLync Pathway Analysis software using the following formula: (k/m)/ (*n*/N). k = the number of significant metabolites per pathway; m = the total number of detected metabolites per pathway; *n* = the number of significant metabolites in the study; and N = the total number of detected metabolites in the study.

**Table 2:** Pathway enrichment scores for each pathway identified in maternal liver. Pathways with enrichment scores >1 have more metabolites with statistically significant fold changes compared to all other pathways within the study.

Sub-Pathway	Pathway Enrichment Score ^1^		
	Gain	OCM	Gain × OCM
Lysophospholipid	1.19	0.59	1.06
Sphingomyelins	1.92	1.04	0.62
Phosphatidylcholine (PC)	1.25	0.24	0.00
Diacylglycerol	1.43	1.01	1.80
Plasmalogen	1.26	0.50	0.90
Monoacylglycerol	1.19	1.43	3.41
Phosphatidylethanolamine (PE)	1.13	0.50	0.00
Phosphatidylinositol (PI)	1.81	0.00	0.00
Fatty Acid Metabolism (Acyl Carnitine, Long-Chain Saturated)	2.26	0.00	0.00
Fatty Acid, Dicarboxylate	0.59	0.34	0.60
Long-Chain Polyunsaturated Fatty Acid (n3 and n6)	0.71	0.00	1.71
Phospholipid Metabolism	1.36	1.81	0.00
Lysoplasmalogen	1.94	1.29	2.32
Fatty Acid Metabolism (Acyl Carnitine, Hydroxy)	1.70	1.13	2.03
Fatty Acid, Monohydroxy	0.47	0.00	0.00
Fatty Acid Metabolism (Acyl Carnitine, Polyunsaturated)	1.13	0.91	1.62
Fatty Acid Metabolism (Acyl Carnitine, Monounsaturated)	1.62	1.29	2.32
Phosphatidylserine (PS)	1.62	2.59	0.00
Dihydrosphingomyelins	1.81	0.00	0.00
Ceramides	0.91	0.00	0.00
Fatty Acid Metabolism (Acyl Glycine)	0.91	0.00	0.00
Long-Chain Monounsaturated Fatty Acid	1.29	0.00	0.00
Endocannabinoid	0.68	0.91	8.10
Fatty Acid Metabolism (Acyl Carnitine, Medium-Chain)	1.70	0.00	0.00
Eicosanoid	0.85	0.00	0.00
Fatty Acid Metabolism (Acyl Carnitine, Dicarboxylate)	2.26	0.00	0.00
Phosphatidylglycerol (PG)	1.36	0.00	0.00
Glycosyl PE	1.36	1.81	3.24
Sterol	1.70	0.00	0.00
Fatty Acid Metabolism (also BCAA Metabolism)	0.97	1.29	0.00
Fatty Acid, Dihydroxy	0.91	0.00	0.00
Secondary Bile Acid Metabolism	0.41	1.65	2.95
Long-Chain Saturated Fatty Acid	0.65	0.00	0.00
Fatty Acid, Branched	0.91	0.00	3.24
Hexosylceramides (HCER)	2.26	0.00	0.00
Fatty Acid Metabolism (Acyl Carnitine, Short-Chain)	2.26	0.00	0.00
Primary Bile Acid Metabolism	0.19	1.51	0.00
Sphingolipid Synthesis	0.57	2.26	0.00
Carnitine Metabolism	1.13	0.00	0.00
Mevalonate Metabolism	0.75	0.00	0.00
Glycerolipid Metabolism	0.75	0.00	0.00
Fatty Acid Metabolism	0.57	2.26	4.05
Fatty Acid, Oxidized	2.26	0.00	0.00
Docosanoid	1.13	0.00	0.00
Medium-Chain Fatty Acid	2.26	0.00	0.00
Dihydroceramides	1.13	0.00	0.00
Lactosylceramides (LCER)	2.26	0.00	0.00
Fatty Acid, Amino	1.13	4.53	0.00
Inositol Metabolism	1.13	4.53	0.00
Progestin Steroids	2.26	0.00	0.00
Fatty Acid Metabolism (Acyl Choline)	0.00	0.00	0.00
Sphingosines	0.00	3.02	0.00
Ketone Bodies	0.00	0.00	0.00
Pregnenolone Steroids	0.00	0.00	0.00
Fatty Acid Synthesis	0.00	0.00	0.00

^1^ Pathway enrichment was calculated within the MetaboLync Pathway Analysis software using the following formula: (k/m)/ (*n*/N). k = the number of significant metabolites per pathway; m = the total number of detected metabolites per pathway; *n* = the number of significant metabolites in the study; and N = the total number of detected metabolites in the study.

**Table 3:** Metabolites involved in selected lipid metabolism sub-pathways in fetal liver.

Sub-Pathway	Biochemical Name	Two-Way ANOVA Main Effects ^1^			Two-Way ANOVA Contrasts ^2^					
		Gain	Supp.	Gain × Supp.	CON + OCM	RES + OCM	RES − OCM	RES + OCM	RES	+OCM
					CON − OCM	RES − OCM	CON − OCM	CON + OCM	CON	−OCM
Fatty Acid Metabolism (Acyl Carnitine, Monounsaturated)	cis-4-decenoylcarnitine (C10:1)	**0.04**	0.21	0.57	1.22	1.17	0.8	**0.77**	**0.79**	1.2
Fatty Acid Metabolism (Acyl Carnitine, Polyunsaturated)	dihomo-linoleoylcarnitine (C20:2)	0.29	0.74	**0.04**	0.79	**1.43**	0.88	**1.59**	1.24	1.11
	docosatrienoylcarnitine (C22:3)	0.44	0.69	**0.04**	0.65	1.42	0.85	**1.87**	1.36	1.04
Fatty Acid Metabolism (Acyl Carnitine, Dicarboxylate)	pimeloylcarnitine/3-methyladipoylcarnitine (C7-DC)	**0.01**	0.2	0.56	1.12	1.34	**0.57**	0.68	**0.63**	1.23
Plasmalogen	1-palmityl-2-palmitoyl-GPC (O-16:0/16:0)	**0.02**	0.53	0.81	1.03	1.02	**1.11**	1.1	**1.11**	1.03
Lysoplasmalogen Monoacylglycerol	1-palmityl-GPC (O-16:0)	0.53	**0.1**	**0.01**	**1.48**	0.9	**1.33**	**0.8**	1.07	1.19
	1-stearyl-GPC (O-18:0)	0.53	0.17	**0.03**	**1.43**	0.93	1.14	**0.74**	0.94	1.18
	1-myristoylglycerol (14:0)	0.17	**0.05**	0.78	0.67	**0.51**	1.58	1.2	1.39	**0.59**
	1-pentadecanoylglycerol (15:0)	0.44	**0.04**	0.64	0.73	**0.54**	1.41	1.04	1.23	**0.64**
	1-linoleoylglycerol (18:2)	0.81	**0.04**	0.62	0.76	**0.48**	1.47	0.92	1.2	**0.62**
	1-docosahexaenoylglycerol (22:6)	0.29	**0.02**	0.73	0.67	**0.43**	1.78	1.14	1.46	**0.55**
	2-myristoylglycerol (14:0)	0.25	**0.04**	0.89	0.58	0.58	1.38	1.38	1.38	0.58
	2-docosahexaenoylglycerol (22:6)	0.47	**0.04**	0.95	0.63	0.48	1.54	1.17	1.36	0.56
	1-heptadecenoylglycerol (17:1)	0.21	**0.02**	0.4	**0.49**	0.54	1.3	1.43	1.37	**0.52**
	2-heptadecenoylglycerol (17:1)	0.22	**0.03**	0.33	**0.52**	0.62	1.23	1.46	1.35	**0.57**
Diacylglycerol	linoleoyl-docosahexaenoyl-glycerol (18:2/22:6) [2]	0.77	**0.03**	0.61	0.81	**0.63**	1.04	0.81	0.93	**0.72**
Hexosylceramides (HCER)	glycosyl-N-stearoyl-sphingosine (d18:1/18:0)	**0.02**	0.81	0.77	0.94	1.04	1.2	**1.33**	**1.27**	0.99
Sphingomyelins	sphingomyelin (d18:2/23:1)	0.28	**0.03**	0.64	**0.77**	0.86	1.05	1.17	1.11	**0.82**

^1^ Blue-shaded cells indicate significant (*p* ≤ 0.05) effect and light blue indicates 0.05 < *p* < 0.10 effect. Non-colored text and cells indicate mean values that are not significantly different within the comparison. ^2^ Red- and green-shaded cells indicate *p* ≤ 0.05 (red = mean values are significantly greater for the comparison; green = mean values are significantly lower for the comparison). Light red- and light green-shaded cells indicate 0.10 > *p* > 0.05 (light red = mean values tend to be greater for the comparison; light green = mean values tend to be lower for the comparison).

**Table 4:** Metabolites involved in selected lipid metabolism sub-pathways in maternal liver.

Sub-Pathway	Biochemical Name	Two-Way ANOVA Main Effects ^1^			Two-Way ANOVA Contrasts ^2^					
		Gain	Supp.	Gain × Supp.	CON + OCM	RES + OCM	RES − OCM	RES + OCM	RES	+OCM
					CON − OCM	RES − OCM	CON − OCM	CON + OCM	CON	−OCM
Fatty Acid Metabolism (Acyl Carnitine, Short-Chain)	acetylcarnitine (C2)	**0.01**	0.42	0.57	1	0.84	**2.02**	**1.71**	**1.87**	0.92
	isocaproylcarnitine	**0.01**	0.64	0.92	1.05	1.04	**2**	**1.97**	**1.99**	1.05
Fatty Acid Metabolism (Acyl Carnitine, Medium-Chain)	hexanoylcarnitine (C6)	**0.01**	0.85	0.9	1.02	1	**2.01**	**1.96**	**1.99**	1.01
	2-methylhexanoylcarnitine	**0.01**	0.58	0.57	0.94	1.18	**1.52**	**1.89**	**1.71**	1.06
	laurylcarnitine (C12)	**0.01**	0.2	0.69	1.37	1.14	**2**	**1.67**	**1.84**	1.26
Fatty Acid Metabolism (Acyl Carnitine, Long-Chain Saturated)	myristoylcarnitine (C14)	**0.01**	0.83	0.94	0.97	0.96	**1.8**	**1.79**	**1.8**	0.97
	pentadecanoylcarnitine (C15)	**0.01**	0.9	0.79	0.91	1.09	**2.34**	**2.79**	**2.57**	1
	palmitoylcarnitine (C16)	**0.01**	0.7	0.22	1.05	0.86	**2.01**	**1.65**	**1.83**	0.96
	margaroylcarnitine (C17)	**0.01**	0.62	0.77	1.07	1.06	**2.7**	**2.68**	**2.69**	1.07
	stearoylcarnitine (C18)	**0.01**	0.32	0.26	1.01	0.83	**2.03**	**1.66**	**1.85**	0.92
	arachidoylcarnitine (C20)	**0.01**	0.6	0.45	0.87	1	**1.78**	**2.06**	**1.92**	0.94
	behenoylcarnitine (C22)	**0.01**	0.71	0.22	0.79	1.12	**1.89**	**2.68**	**2.29**	0.96
	lignoceroylcarnitine (C24)	**0.01**	0.67	0.82	0.96	0.87	**2.83**	**2.56**	**2.7**	0.92
Fatty Acid Metabolism (Acyl Carnitine, Monounsaturated)	5-dodecenoylcarnitine (C12:1)	**0.01**	0.98	0.69	1.06	0.93	**3.13**	**2.77**	**2.95**	1
	palmitoleoylcarnitine (C16:1)	**0.01**	0.54	0.35	1.12	0.97	**2.29**	**1.98**	**2.14**	1.05
	oleoylcarnitine (C18:1)	**0.01**	0.54	0.58	0.96	0.88	**1.85**	**1.69**	**1.77**	0.92
	eicosenoylcarnitine (C20:1)	**0.01**	**0.01**	**0.01**	0.99	**0.54**	**2.24**	1.23	**1.74**	**0.77**
	erucoylcarnitine (C22:1)	**0.01**	0.51	0.36	0.7	1.02	**2.72**	**3.99**	**3.36**	0.86
Fatty Acid Metabolism (Acyl Carnitine, Polyunsaturated)	linoleoylcarnitine (C18:2)	**0.01**	0.9	0.46	1.06	0.91	**1.73**	**1.47**	**1.6**	0.99
	linolenoylcarnitine (C18:3)	**0.01**	0.46	0.48	0.9	0.98	1.25	**1.35**	**1.3**	0.94
	arachidonoylcarnitine (C20:4)	**0.01**	0.56	0.29	1.12	0.98	**1.65**	**1.43**	**1.54**	1.05
	adrenoylcarnitine (C22:4)	**0.01**	0.76	0.97	0.98	0.95	**0.72**	**0.71**	**0.72**	0.97
Fatty Acid Metabolism (Acyl Carnitine, Dicarboxylate)	adipoylcarnitine (C6-DC)	**0.01**	0.43	0.59	0.88	0.94	**2.35**	**2.52**	**2.44**	0.91
	pimeloylcarnitine/3-methyladipoylcarnitine (C7-DC)	**0.01**	0.92	0.72	1.02	0.95	**2.01**	**1.87**	**1.94**	0.99
	suberoylcarnitine (C8-DC)	**0.01**	0.4	0.48	0.91	0.97	**1.75**	**1.88**	**1.82**	0.94
Fatty Acid Metabolism (Acyl Carnitine, Hydroxy)	(R)-3-hydroxybutyrylcarnitine	**0.01**	0.39	0.83	1.1	1.05	**1.39**	**1.33**	**1.36**	1.08
	(S)-3-hydroxybutyrylcarnitine	**0.01**	0.32	0.47	0.95	0.78	**1.62**	1.33	**1.48**	0.87
	3-hydroxyhexanoylcarnitine (1)	**0.01**	0.58	0.69	0.79	0.94	**1.62**	**1.93**	**1.78**	0.87
	3-hydroxypalmitoylcarnitine	**0.01**	0.82	0.5	1.16	0.88	**2.27**	**1.72**	**2**	1.02
	3-hydroxyoleoylcarnitine	**0.01**	0.86	0.52	1.06	0.92	**2.24**	**1.94**	**2.09**	0.99
Plasmalogen	1-palmityl-2-palmitoyl-GPC (O-16:0/16:0)	**0.01**	0.43	0.89	0.95	0.92	**2.02**	**1.97**	**2**	0.94
	1-palmityl-2-stearoyl-GPC (O-16:0/18:0)	**0.01**	0.34	1	0.93	0.93	**1.64**	**1.64**	**1.64**	0.93
	1-palmityl-2-linoleoyl-GPC (O-16:0/18:2)	**0.01**	0.69	0.24	1.11	0.94	**1.36**	1.15	**1.26**	1.03
	1-stearyl-2-arachidonoyl-GPC (O-18:0/20:4)	**0.01**	0.64	0.93	0.96	0.96	**1.64**	**1.64**	**1.64**	0.96
	1-(1-enyl-palmitoyl)-2-linoleoyl-GPE (P-16:0/18:2)	**0.01**	0.85	0.71	0.99	0.91	**0.56**	**0.52**	**0.54**	0.95
	1-(1-enyl-palmitoyl)-2-palmitoyl-GPC (P-16:0/16:0)	**0.01**	0.66	0.83	0.98	0.96	**1.51**	**1.47**	**1.49**	0.97
	1-(1-enyl-palmitoyl)-2-arachidonoyl-GPE (P-16:0/20:4)	**0.01**	0.47	0.3	0.89	1.04	1.15	**1.34**	**1.25**	0.97
	1-(1-enyl-stearoyl)-2-oleoyl-GPE (P-18:0/18:1)	**0.01**	0.58	0.3	1.1	0.97	**1.55**	**1.36**	**1.46**	1.04
	1-(1-enyl-stearoyl)-2-arachidonoyl-GPE (P-18:0/20:4)	**0.01**	0.66	0.26	1.08	0.97	**1.56**	**1.42**	**1.49**	1.03
Lysoplasmalogen	1-palmityl-GPC (O-16:0)	**0.01**	0.92	0.51	0.92	1.13	**2.27**	**2.79**	**2.53**	1.03
	1-stearyl-GPC (O-18:0)	**0.01**	0.38	0.78	0.86	0.96	**2.07**	**2.32**	**2.2**	0.91
	1-stearyl-GPE (O-18:0)	**0.01**	0.43	0.61	1.02	1.15	**1.72**	**1.93**	**1.83**	1.09
	1-(1-enyl-palmitoyl)-GPE (P-16:0)	**0.01**	0.57	0.28	1.02	0.85	**1.49**	1.23	**1.36**	0.94
	1-(1-enyl-oleoyl)-GPE (P-18:1)	**0.01**	0.48	0.22	1.05	0.8	**1.68**	1.29	**1.49**	0.93
Monoacylglycerol	1-myristoylglycerol (14:0)	**0.01**	0.22	0.17	0.98	**0.54**	**2.35**	1.3	**1.83**	0.76
	1-palmitoleoylglycerol (16:1)	**0.01**	0.24	0.38	0.84	0.45	**4.93**	**2.65**	**3.79**	0.65
	1-oleoylglycerol (18:1)	**0.01**	0.44	0.59	1.15	0.59	**4.9**	**2.53**	**3.72**	0.87
	1-linoleoylglycerol (18:2)	**0.05**	0.8	**0.01**	1.45	**0.53**	**2.63**	0.97	**1.8**	0.99
	1-docosahexaenoylglycerol (22:6)	**0.1**	**0.04**	**0.09**	0.92	**0.51**	**1.79**	0.99	**1.39**	**0.72**
	2-myristoylglycerol (14:0)	0.81	**0.01**	0.66	**0.63**	**0.56**	1.02	0.91	0.97	**0.6**
	2-oleoylglycerol (18:1)	**0.01**	0.15	0.42	0.91	0.6	**2.91**	**1.91**	**2.41**	0.76
	2-linoleoylglycerol (18:2)	**0.01**	0.38	**0.02**	1.22	**0.56**	**2.31**	1.06	**1.69**	0.89
	2-arachidonoylglycerol (20:4)	**0.02**	0.63	**0.03**	1.31	**0.63**	**2.23**	1.06	**1.65**	0.97
	2-docosahexaenoylglycerol (22:6)	**0.01**	**0.01**	**0.03**	0.94	**0.58**	**1.68**	1.04	**1.36**	**0.76**
	1-heptadecenoylglycerol (17:1)	**0.01**	0.35	**0.08**	1.39	**0.41**	**6.17**	1.8	**3.99**	0.9
	2-heptadecenoylglycerol (17:1)	**0.01**	0.38	0.34	1.02	0.56	**3.19**	**1.74**	**2.47**	0.79
Diacylglycerol	diacylglycerol (12:0/18:1, 14:0/16:1, 16:0/14:1) [2]	**0.01**	0.11	0.59	0.57	0.81	**4.55**	**6.42**	**5.49**	0.69
	diacylglycerol (14:0/18:1, 16:0/16:1) [2]	**0.01**	0.69	0.66	0.86	0.86	**3.53**	**3.53**	**3.53**	0.86
	diacylglycerol (16:1/18:2 [2], 16:0/18:3 [1])	**0.01**	0.95	0.77	0.98	0.95	**3.26**	**3.15**	**3.21**	0.97
	palmitoyl-oleoyl-glycerol (16:0/18:1) [1]	**0.01**	0.78	0.78	1	0.91	**2.24**	**2.03**	**2.14**	0.96
	palmitoyl-oleoyl-glycerol (16:0/18:1) [2]	**0.01**	0.66	0.44	1	0.87	**3.01**	**2.61**	**2.81**	0.94
	palmitoyl-linoleoyl-glycerol (16:0/18:2) [2]	**0.01**	0.28	0.29	1.02	0.83	**2.13**	**1.73**	**1.93**	0.93
	palmitoyl-linolenoyl-glycerol (16:0/18:3) [2]	**0.01**	0.21	0.65	0.76	0.85	**1.98**	**2.23**	**2.11**	0.81
	palmitoyl-arachidonoyl-glycerol (16:0/20:4) [2]	**0.01**	0.4	0.32	1.02	0.88	**1.89**	**1.63**	**1.76**	0.95
	palmitoyl-docosahexaenoyl-glycerol (16:0/22:6) [2]	**0.01**	0.45	0.65	0.99	0.9	**1.42**	**1.29**	**1.36**	0.95
	oleoyl-linoleoyl-glycerol (18:1/18:2) [1]	**0.01**	0.6	0.19	1.28	0.74	**3.01**	1.74	**2.38**	1.01
	oleoyl-linoleoyl-glycerol (18:1/18:2) [2]	**0.01**	0.63	0.81	0.98	0.8	**2.44**	**2.01**	**2.23**	0.89
	stearoyl-arachidonoyl-glycerol (18:0/20:4) [2]	**0.01**	0.39	0.17	1.2	0.97	**1.49**	1.2	**1.35**	1.09
	oleoyl-arachidonoyl-glycerol (18:1/20:4) [2]	**0.01**	0.92	0.31	1.12	0.94	**1.46**	1.23	**1.35**	1.03
	linoleoyl-arachidonoyl-glycerol (18:2/20:4) [2]	**0.02**	0.45	0.21	1.1	0.67	**2.2**	1.33	**1.77**	0.89
Hexosylceramides (HCER)	glycosyl-N-palmitoyl-sphingosine (d18:1/16:0)	**0.01**	0.65	0.5	1.02	0.92	**1.57**	**1.42**	**1.5**	0.97
	glycosyl-N-stearoyl-sphingosine (d18:1/18:0)	**0.01**	0.38	0.13	1.07	**0.82**	**1.94**	**1.49**	**1.72**	0.95
Lactosylceramides (LCER)	lactosyl-N-palmitoyl-sphingosine (d18:1/16:0)	**0.01**	0.33	0.45	0.92	0.85	**1.35**	1.26	**1.31**	0.89
Dihydrosphingomyelins	myristoyl dihydrosphingomyelin (d18:0/14:0)	**0.01**	0.5	0.74	0.91	0.94	**1.83**	**1.88**	**1.86**	0.93
	palmitoyl dihydrosphingomyelin (d18:0/16:0)	**0.01**	0.62	0.22	1.13	0.95	**1.56**	**1.32**	**1.44**	1.04
	behenoyl dihydrosphingomyelin (d18:0/22:0)	**0.01**	0.43	0.37	1.16	0.98	**1.67**	**1.42**	**1.55**	1.07
	sphingomyelin (d18:0/20:0, d16:0/22:0)	**0.01**	0.39	0.18	1.24	0.93	**1.62**	1.22	**1.42**	1.09
Sphingomyelins	palmitoyl sphingomyelin (d18:1/16:0)	**0.02**	0.3	0.46	0.95	0.99	1.05	**1.09**	**1.07**	0.97
	behenoyl sphingomyelin (d18:1/22:0)	**0.01**	0.62	0.91	0.99	0.98	**1.32**	**1.31**	**1.32**	0.99
	tricosanoyl sphingomyelin (d18:1/23:0)	**0.01**	0.83	0.99	1	0.99	**1.29**	**1.27**	**1.28**	1
	sphingomyelin (d18:2/18:1)	**0.01**	0.35	0.24	1.23	0.96	**2.09**	**1.63**	**1.86**	1.1
	sphingomyelin (d18:2/23:1)	**0.02**	0.4	0.92	0.84	0.89	1.38	**1.47**	**1.43**	0.87
	sphingomyelin (d17:1/14:0, d16:1/15:0)	**0.04**	0.38	0.98	0.94	0.96	0.88	0.89	0.89	0.95
	sphingomyelin (d18:2/14:0, d18:1/14:1)	**0.01**	**0.03**	0.42	**0.79**	0.9	**0.64**	**0.73**	**0.69**	**0.85**
	sphingomyelin (d17:1/16:0, d18:1/15:0, d16:1/17:0)	**0.01**	**0.1**	0.74	0.92	0.95	**1.18**	**1.22**	**1.2**	0.94
	sphingomyelin (d17:2/16:0, d18:2/15:0)	**0.01**	0.4	0.31	0.84	1.04	**0.53**	**0.66**	**0.6**	0.94
	sphingomyelin (d18:2/16:0, d18:1/16:1)	**0.03**	0.68	0.47	0.96	1.01	**0.9**	0.95	**0.93**	0.99
	sphingomyelin (d18:1/17:0, d17:1/18:0, d19:1/16:0)	**0.01**	0.35	0.35	0.88	0.98	**0.69**	**0.77**	**0.73**	0.93
	sphingomyelin (d18:1/19:0, d19:1/18:0)	**0.01**	0.24	0.2	1.01	**0.86**	**1.44**	**1.22**	**1.33**	0.94
	sphingomyelin (d18:1/20:0, d16:1/22:0)	**0.01**	0.17	0.16	1	**0.9**	**1.34**	**1.21**	**1.28**	0.95
	sphingomyelin (d18:1/20:1, d18:2/20:0)	**0.03**	0.87	0.79	1.03	0.99	0.78	**0.76**	**0.77**	1.01
	sphingomyelin (d18:1/21:0, d17:1/22:0, d16:1/23:0)	**0.01**	**0.04**	0.77	0.93	**0.91**	**1.41**	**1.39**	**1.4**	**0.92**
	sphingomyelin (d18:2/21:0, d16:2/23:0)	**0.01**	0.26	0.35	0.96	0.82	**1.63**	**1.39**	**1.51**	0.89
	sphingomyelin (d18:1/22:1, d18:2/22:0, d16:1/24:1)	**0.01**	0.15	0.22	0.99	**0.83**	**1.42**	1.2	**1.31**	0.91
	sphingomyelin (d18:2/23:0, d18:1/23:1, d17:1/24:1)	**0.01**	0.29	0.72	0.93	0.95	**1.25**	**1.28**	**1.27**	0.94
	sphingomyelin (d18:1/24:1, d18:2/24:0)	**0.01**	0.19	0.59	0.96	0.92	**1.31**	**1.25**	**1.28**	0.94
	sphingomyelin (d18:2/24:1, d18:1/24:2)	**0.01**	0.95	0.38	1.06	0.95	**1.29**	**1.15**	**1.22**	1.01
	sphingomyelin (d18:1/25:0, d19:0/24:1, d20:1/23:0, d19:1/24:0)	**0.01**	0.78	0.69	1.01	0.97	**0.84**	**0.81**	**0.83**	0.99
Sterol	cholesterol	**0.01**	0.2	0.83	0.97	0.96	**1.16**	**1.14**	**1.15**	0.97
	4-cholesten-3-one	**0.04**	0.97	0.93	1	0.95	1.26	1.2	1.23	0.98
	7-hydroxycholesterol (alpha or beta)	**0.01**	0.59	0.61	1.01	1.43	1.67	**2.38**	**2.03**	1.22
Secondary Bile Acid Metabolism	taurodeoxycholate	0.56	**0.03**	0.95	1.91	**1.43**	1.09	0.82	0.96	**1.67**
	glycoursodeoxycholate	**0.01**	0.15	**0.02**	**0.35**	1.52	**2.15**	**9.29**	**5.72**	0.94
	tauroursodeoxycholate	**0.01**	0.23	0.3	1	1.45	**2.86**	**4.13**	**3.5**	1.23
	glycocholenate sulfate	0.2	**0.01**	0.79	**0.67**	**0.73**	1.09	1.18	1.14	**0.7**
	taurocholenate sulfate	0.14	0.14	**0.05**	**1.97**	0.91	1.23	**0.57**	0.9	1.44

^1^ Blue-shaded cells indicate significant (*p* ≤ 0.05) effect and light blue indicates 0.05 < *p* < 0.10 effect. Non-colored text and cells indicate mean values are not significantly different within the comparison. ^2^ Red- and green-shaded cells indicate *p* ≤ 0.05 (red = mean values are significantly greater for the comparison; green = mean values are significantly lower for the comparison). Light red- and light green-shaded cells indicate 0.10 > *p* > 0.05 (light red = mean values tend to be greater for the comparison; light green = mean values tend to be lower for the comparison).

## Data Availability

The data presented in this study are available on request from the corresponding author.

## Supplementary Materials

The following supporting information can be downloaded at: https://www.mdpi.com/article/10.3390/metabo15050302/s1, Figure S1: Boxplots for the comparison of concentrations of different treatment groups among the significant lipid metabolites; File S1: *p*-values, fold changes, mean values, and percentage-filled values for all the metabolites; File S2: Batch-norm-imputed data for all metabolites involved in lipid pathways.

## Abbreviations

ADG	Average daily gain
CON	Control
KEGG	Kyoto Encyclopedia of Genes and Genomes
OCM	One-carbon metabolism
PCA	Principal component analysis
MAG	Monoacylglycerol
S1P	Sphingosine-1-phosphate
FAO	Fatty acid β-oxidation
LCER	Lactosylceramides
UPLC	Ultra-performance liquid chromatography
VLDL	Very-low-density lipoprotein
SAM	S-Adenosylmethionine
HCER	Hexosylceramides
RES	Restricted
